# Supplementary material for: Analysis of lithium trade patterns and influencing factors in the regions along the "Belt and Road"
Source: PLoS One. 2024 Aug 21;19(8):e0307321. doi: 10.1371/journal.pone.0307321 (PMC11338446; doi:10.1371/journal.pone.0307321)
Supplement: Attachment — Submitted filename: Response to Reviewers.docx [file pone.0307321.s002.docx]

*Responds of* *Analysis of Lithium Trade Patterns and Influencing Factors in the Regions Along the "Belt and Road"*

Dear academic editor and reviewers,

We appreciate your comments and suggestions on our manuscript. These comments are valuable and helpful to improve our manuscript. According to the comments, revision has been made for the manuscript. The editor and reviews comments are quoted in bold font and numbered for convenience. Our response is given in normal font and the changes or additions we made to the manuscript are highlighted in blue. We hope to make it better if the reviewers or the editor give us any comments in the next communication.

The followings are point-to-point response to the reviewers’ comments.

Sincerely,

Peng Du

Assistant Professor

Liaoning Normal University

Academic Editor:

**Comments:** It's crucial that the authors thoroughly address all concerns raised by the reviewers to ensure the integrity and quality of their work. Addressing these concerns not only strengthens the credibility of the research but also demonstrates a commitment to transparency and rigor in the scientific process. By carefully considering and responding to each reviewer's feedback, the authors can enhance the clarity, validity, and overall impact of their study.

**Response：**We are grateful to the academic editor and the reviewers for their feedback, we have made efforts to ensure the integrity and quality of our manuscript as much as possible. We have considered these comments carefully and responded to each reviewer’s feedback sincerely.

**Dear reviewers:**

Thank you very much for your comments and professional advice. These opinions help to improve academic rigor of our article. Based on your suggestions and requests, we have made corrected modifications on the revised manuscript. We hope that our work can be improved again. Furthermore, we would like to show the details as follows:

**Response to Reviewer #1’s comments:**

**Reviewer #1:** This study studies the trade patterns and influencing factors of lithium in the regions along the "Belt and Road". The structure of the article is clear and logical. However, here are some comments for the author's reference:

**Comments 1:** Some sentences in the abstract are lengthy, which can be further simplified to improve readability.

**Response 1：**Thank you very much for your advice. According your advice, we have made some simplifications for this essay’s language. Not only in the abstract section, but we have simplified the language in other parts of the text as well, and we have marked them in blue.

**Abstract:** Lithium has broad applications in several emerging industries and fields, including high energy batteries, energy storage, aerospace, and controlled nuclear reactions. Currently, the discrepancy between the supply and demand for lithium resources increases, and its distribution is uneven. Within the framework of the "Belt and Road" Initiative, the lithium trade pattern evolves constantly. However, the trade pattern of lithium in the nations along the "Belt and Road" is likely to face substantial repercussions in modern world of unilateral protectionism and geopolitical conflicts. Taking the social network analysis approach as a tool, this study first examines the characteristics of the lithium trade network structure as it has evolved over the years in the Belt and Road countries, from 2000 to 2022. Additionally, this study uses the quadratic assignment problem approach to analyze the factors influencing the evolution of the lithium trade network. The study shows that: (1) The spatial patterns of import and export trade network of lithium in countries along the route has a certain path dependence. And the market is mainly concentrated in East Asia, Central and Eastern Europe, South America and Southeast Asia. (2) The network density of the countries along the route has increased year after year, but it remains low. And the fluctuation of the network's reciprocity has increased, with a huge magnitude of variation. The number of core countries in the network has decreased over time, but the core-periphery structure has stayed largely steady. China, Chile, and South Korea are the network's main node countries. (3) Regarding the influencing factors, the differences in economic and technological development between these countries have a beneficial impact on the formation of lithium trade; whether or not regional trade agreements have been signed, the differences about average tax rates for mineral products, bordering countries, and similar languages and cultures are all conducive to the establishment of close trade links. The contribution of this essay is of paramount importance for understanding different countries’ role along the Belt and Road in the lithium trade network pattern, and promoting regional trade cooperation.

**Other parts’ simplifications are as details:**

1. **For the continuity and comparability of academic research, some contemporary scholars take the traditional 65 nations within the B&R as the object of research.**

**2.** in 2019, the outbreak of the COVID-19 epidemic, a public health event, had tremendous impacts and challenges for countries around the world;

3. **This essay utilizes import and export data from the UN Comtrade Database (https://comtradeplus.un.org/) for lithium, such as lithium hydroxide and lithium oxides, lithium carbonate and lithium perfluorooctyl sulfonate, from nations and areas across the B&R between 2000 and 2022.**

**4. After verifying the customs HS code, the definition of specific lithium and its corresponding customs code are shown in Table 1.**

**5. The social network is essentially composed of nodes and edges, and it represents a collection of social players and their connections.**

**6. This study primarily selects the data of import countries, export countries and trade value, and takes the lithium trade nations throughout the B&R as nodes, as well as the global flows of trade within the route as edges, respectively, to construct the lithium trade network of oriented weighted and unoriented unweighted from 2000 to 2022.**

**7. Reciprocity is defined as the fraction of relationships in the structure that allow for two-way trade to the total number of relationships established in the network, which measures the degree of connectedness between the two nodes in the network, can be used to determine how stable the two-way commerce in lithium has become.**

**8.** **The UCINET software calculates the core degree for each nation that takes part in the B&R trade network for lithium.**

**9. The connecting lines depict the lithium trade relations between two countries in a particular year.**

**10. From the import perspective, the import pattern of lithium in the countries along the route is highly concentrated, with an obvious pathway dependence.**

**11. From 2000 to 2003, the lithium trade patterns that were initially formed in the countries along the path were formed, and there is not much of a trade volume difference between the countries.**

12. According to the volume of import and export trade, no significant change in trade volume in 2019. While 2019 could have been impacted by COVID-19 outbreak, it was the end of the year and the impact on the economic and trade activities of the countries along the route was minimal.

13. In 2022, despite the trade network appears to be sparse, lithium account for more than10 billion U.S. dollars of total imports.

14. China imported lithium worth 6.7 billion U.S. dollars solely from Chile, constituting more than half of the whole trade volume of countries along the B&R with lithium resources.

15. From an export perspective, lithium’ export in countries within the B&R is relatively concentrated, and the exporting countries are primarily centralized in Chile, China, Italy and other places.

16. Compared to the previous year, Russian Federation has experienced a notable decline in their total import and export trade of lithium in 2022, particularly with several Central and Eastern European countries.

17. Lithium’s export pattern of countries along the B&I.

18. An analysis of the overall lithium trade network in the nations bordering the "Belt and Road".

**Comments 2:** The conclusion section of the abstract should more clearly summarize the main findings and implications of the study. While the current abstract lists the findings, it does not generalize about the implications or implications of the study in a whole.

**Response 2：**Thank you for your advice. In the part of Abstract, we list the findings and try to make it more clearly, and we also try to generalize the implications and main contributions of this study. This revised part can be seen in the manuscript which marked in blue in the revised manuscript or the response for question 1.

**Comments 3:** Authors have included literature review in ‘Introduction’ section related to certain aspects of topic, but it does not seem to have a logical overall flow. Relevant literature review should be done in the line with the following question: What is the limitation about previous studies, and how this paper fills the gap? Your literature review should lead to the specific hypothesis for what you expect to find in targeted area.

**Response 3：**Thank you for your advice. We adjust the relevant literature review according to the limitations, previous studies and this paper how to fill the gap. Changes as follows:

In 2013, Chinese President Xi Jinping proposed the Belt and Road Initiative (BRI); and in 2023, the third Belt and Road Summit on International Cooperation has been held in Beijing. The Belt and Road Cooperation Initiative, a model of South-South cooperation, has produced impressive results in the last ten years in the fields of energy, commerce and economy, building infrastructure, and green development. It has also developed into a significant global public utility and useful platform that has aided in global development and has kept moving in the direction of sustainability, high standards, and improving people's quality of life[1]. In light of the intricate and significant changes occurring in the world today, including the escalation of protectionist inclinations and the persistent rise in uncertainty regarding economic globalization, China has offered a Chinese solution to the global governance system by fostering greater collaboration among the vast majority of developing nations, advancing the economic growth and infrastructure building of the nations along the Belt and Road, and fostering friendship and prosperity. Considering the intricate and significant changes taking place in the contemporary world, the BRI is a major initiative for China to integrate into the Eurasian continent in the new era, and the pattern of energy power shift that characterizes the Belt and Road region is destined to raise major issues[2]. The unstable pattern of international trade and the uneven distribution of key mineral resources resulted in a complex trading dynamic between the nations involved in the Belt and Road, which is not amenable to easy definition and description, forming a complex network structure[3]. In addition, the trading network has a "hybrid" structural framework that exhibits a link design consisting of both hub-and-spoke and fully connected configurations, resulting in high levels of vulnerability and robustness[4].

Regarding social network research, Freeman[5] systematically described the history of the development of social network analysis methods, and argued that many of the concepts in social network theory originated in graph theory, where a set of points consisting of many points and their corresponding lines or edges connecting the points to each other can be depicted on a blank piece of graph paper. Subsequently, Newman[6] found that networks are usually not random and that they have very distinct statistical features, some of which, such as high clustering coefficients and highly skewed degree distributions, are common in networks of all types. Then the world trade network occurred, as discovered by Serrano[7], is a complex network that differs greatly from a traditional random network because of the vertices' degree-correlation features, small-world traits, and prevalence of scale-free distributions. Garlaschelli[8] first defined the world trade network as a network defined by trade relations among countries in the world. Fagiolo[9] used a weighted network approach to study the empirical properties of the network of trade relations among countries and its evolution over time, and found that most countries have weak trade links, but some countries have strong links. Alexandre[10] used a link prediction algorithm based on a current snapshot of the network aimed at predicting its future evolution, and applied it to the international trade data among countries to obtain better results. Ashadun Nobi et al. constructed a minimum spanning tree in world trade based on cross-correlations between time series of different commodities and found that China's influence on the trade network began to emerge after 2004[11]. In recent years, the social network analysis method is widely used in international trade and other fields, and the material, information and spatial flows of international trade are one of the main research contents of world economic geography. Scholars at home and abroad apply this method in the study of trade networks in different fields and commodities, such as Guo Weidong[12] studied the evolution of the spatio-temporal pattern and driving mechanism of global military science and technology trade in 3 aspects, namely, product structure, network pattern and influence mechanism. Qi Wei et al.[13] selected the 2017-2020 global new energy automobile trade data as a sample to study the dynamic evolution characteristics of the trade network. Xiaoliang Jia et al.[14] proposed a novel wavelet-based complex network method, aim to study the evolution characteristics of the integration and diversification for the world's crude oil market, from the structural perspective of global oil prices’ interdependence. He Shengbing et al.[15] used the 54 environmental products’ trade data of the “Belt and Road” countries from 2000 to 2020 to constructed an environmental product trade network, and used the time-indexed random graph model to explore the structural characteristics of the regional environmental product trade network and its evolution mechanism. In addition, some scholars have also utilized social network theory to construct the trade network of nickel ore[16], chromium ore[17] and licorice extract[18] to analyze their trade structure and trade competitiveness.

Regarding the research on lithium, lithium is the lightest metal in nature, with excellent performance, characterized by strong ability to gain and lose electrons and high specific capacity, which is widely used in a number of new technology industries and fields, such as high-energy batteries, energy storage, aerospace, controlled nuclear reactions, and so on[19-20]. Currently, the status of lithium in the international arena is rising, and major economies have increased the importance of mineral resources such as lithium. In 2009, the Japanese government first released the Rare Metals Safeguard Strategy[21]; in 2016, China released the National Mineral Resources Plan (2016-2020), which lists 24 minerals (groups) such as oil, natural gas, and other minerals as strategic minerals[23] ; in 2022, the U.S. Geological Survey published a new list of 50 key minerals, and the Australian government released the 2022 Key Minerals Strategy, with lithium included in the above strategic list[22,24]. However, lithium has a certain economic vulnerability, and its industrial chain can be mainly divided into four levels, upstream products (lithium raw ore), basic lithium products, deep-processed lithium products and downstream products[25]. In recent years, the social network analysis method has also been introduced in lithium resources and products trade research. Chen Guang[26] analyzed the characteristics of global lithium carbonate and lithium hydroxide trade communities’ evolution. Zhu Lili et al.[27-28] analyzed the control power of China in the international trade network of lithium hydroxide and lithium carbonate. Wu Qiaosheng et al.[29] established a global dynamic trade network for lithium products, analyzing the degree of trade diversity and trade influence of China in the global trade network through weighted degree and other indicators. However, most of the above studies focus on the community division of lithium trade or the controlling role of a certain country in the trade network from the global or China’s perspective, and less on the lithium trade pattern evolution and its influencing factors from the Belt and Road perspective, which makes it difficult for the countries along the route to study their relative position and influence in the lithium trade network.

In conclusion, there are currently more abundant international studies related to lithium, and there are also many studies on trade networks. However, some studies have utilized the intensity of access to identify important nodes, but have not taken into account the importance of the nodes associated with them in the trade network. In view of this, this study not only portrays and analyzes the long time series dynamic network evolution of lithium in the countries along the Belt and Road and its influencing factors, but also identifies the key nodes in the trade network of the Belt and Road by using the PageRank (PR) algorithm. The PR algorithm is used to identify key nodes in the “Belt and Road” trade network, and the PR algorithm can rank the search results to prioritize the more important and core web pages or nodes[30]. Unlike the existing literature that uses access degree to identify important nodes in the network, this study attempts to combine the centrality, core-edge structure, and more advanced PR algorithms to explore the evolution of the spatio-temporal pattern of lithium trade in the perspective of the Belt and Road, to analyze the key nodes in the network, and to identify the key nodes in the “Belt and Road” trade network among the countries along the routes. The role and status of the countries along the route in the “Belt and Road” lithium trade network, in order to provide a scientific basis for the selection of trade partners of the countries along the route. It also analyzes the influencing factors of the trade network evolution from five perspectives: economy, system, science and technology, geography and culture, with a view to optimizing the energy trade strategies and policies under the framework of “Belt and Road”, and guaranteeing the high-quality development of China's new energy and new technology industries.

**Comments 4:** Section data is used in part 2.2, which lacks time series information, so it cannot reveal the long-term trend and dynamic changes of lithium trade.

**Response 4：**Part 2.2 shows the data sources and processing of the empirical part of this study. In section 2.1, we select six representative years, 2000, 2003, 2008, 2013, 2019 and 2022, as the cross-section of this study, so as to show the lithium trade pattern evolution of the Belt and Road and the major turning points, so as to reveal the characteristics of the long time series of the lithium trade network pattern. The timeframe of this paper is selected as a cross-section of the study. Although the time range of this paper is 2000-2022, due to space constraints, some more representative years are selected to reveal the dynamic changes of lithium trade. These years are selected for the following reasons: 2000 is the starting year of this study; in 2003, the world economy became extremely fragile and unstable due to the SARS epidemic and currency wars; in 2008, it witnessed the commencement of the world financial crisis, and then the global economic development entered a downturn; in 2013, China put forward the BRI, which profoundly altered the patterns of development for trade and economics worldwide as well as the worldwide allocation of labor; in 2019, the outbreak of the global New Crown Epidemic, a public health event, had tremendous impacts and challenges for countries around the world; in 2022, the Russia-Ukraine conflict had an unprecedented geopolitical impact on the B&R. In light of this, this study selects six representative years, specifically 2000, 2003, 2008, 2013, 2019 and 2022, as the cross-section sample. This decision aims to showcase the evolution of lithium trade development in the B&R and the significant turning points.

**Comments 5:** The empirical part can enrich some methods, such as principal component analysis and factor analysis. Further analysis of the basic lithium product trade network pattern of countries along the route.

**Response 5：**Thank you for your advice. In the empirical part, the network density, core periphery structure, and block model in social network analysis are used to present the overall characteristics of the Belt and Road lithium trade network, the node strength is measured by centrality and PageRank algorithm to portray the characteristics of the different nodes in the trade network, and the changes in the lithium trade network pattern of the countries along the Belt and Road are presented by ArcGIS software. In the empirical part of the influencing factors, the study adopts the quadratic assignment procedure method, which is able to reflect the correlation and regression relationship between relational data. This method is based on the substitution of matrix data, which gives the correlation coefficient between two matrices by comparing the corresponding grid values of each square matrix, and at the same time, the coefficients are subjected to nonparametric tests. QAP regression, on the other hand, studies the regression relationship between multiple factors or matrices and a matrix, and evaluates the significance of the determined coefficients, which is a good method that can exclude the false structural relationship.

**Comments 6:** Some specific measures can be proposed in the conclusion part.

**Response 6：**We completely agree with your comment. According to your suggestion, we have added an extra section in the conclusion section, which gives relevant countermeasure recommendations for the Belt and Road Initiative countries (China) and the Belt and Road participating countries respectively. The details are as follows:

6.2 Suggestions

6.2.1 Suggestions for Belt and One Road Initiative country (China)

(1) Grasp the development opportunities of the Belt and Road Initiative, China should actively discuss and promote the establishment of a strategic cooperation mechanism for lithium resources in countries along the Belt and Road Initiative. The lithium resources governance system is in the early stages of development, with no governance core and authority, and a lack of global coordination mechanisms. China should actively promote the coordination of interests between resource countries and consumer countries, establish lithium industry chain organizations, promote the formation of a global consensus, and put forward a global program for the sustainable supply of green lithium resources.

(2) China should strengthen the degree of domestic lithium resources exploration and development, strengthen the technological innovation of lithium extraction from salt lakes, and increase the investment in the search for lithium salt lakes, lithium pyroxene and lithium mica. Meanwhile, we should also actively build a safe, stable and diversified supply system of lithium resources along the Belt and Road. Strengthen diplomatic relations with the South American “lithium triangle”, enhance the level of strategic cooperation, the use of high-level diplomatic means to create a favorable international investment environment, technology and industrial investment in exchange for local lithium resources. Enhance China's lithium resources in the domestic and international double cycle the safety and security capacity of China's lithium resources in the domestic and international double cycle.

(3) China should improve its foreign-related legal service system. Enterprises not only have to consider industrial, technological, financial and other aspects of risk factors, but also face the host country as well as other countries of the political and social risk of the test. In order to effectively protect the safety of private enterprises investment in overseas minerals, China needs to establish a sound international commercial tribunal and joint arbitration mechanism to provide a trustworthy and dependable dispute resolution platform for China's overseas investment entities. Meanwhile, it should help enterprises carry out compliance training for overseas investment and enhance the comprehensive competitiveness of Chinese enterprises.

6.2.2 Suggestions for Belt and Road participating countries (other countries)

(1) Core countries such as Chile, Argentina and Russia should actively promote the extension of their export markets to Southeast Asia, South Asia, Eastern Europe and Africa, so as to promote the realization of mutual benefits and interconnection of the Belt and Road lithium trade network. Core countries and edge countries should maintain stable relations with the original trading countries on the basis of active development of trade with other countries, with more countries to negotiate free trade agreements, investment protection agreements, reduce trade costs and improve the stability of the network.

(2) Belt and Road countries need to work together with the initiative country (China) to study the creation of rules, enhance the Belt and Road Initiative's global issue-setting and rule-making capabilities, and promote the transformation of the Belt and Road Initiative from commodity-factor flow-type opening to rule system-type opening. The co-construction countries will strengthen the construction of multilateral cooperation platforms for lithium resources, and enhance inter-regional cooperation around the Middle East, Asia-Pacific, Russia-Central Asia, Latin America, Africa and other regions according to their resource endowments.

(3) Actively participate in the construction of “Six Corridors, Six Roads, Multiple Countries and Multiple Ports” to improve the logistics accessibility of the countries along the routes, and build a modern comprehensive transportation system. Poland and Hungary, as the important gateways for China-EU liner to enter the European Union, should grasp the role as important nodes in the transportation network of the Belt and Road, and deepen economic and trade cooperation with China and other Asian countries. As an important carrier of the “Belt and Road” connectivity, China-EU liner maintains the stability and smoothness of the international industrial chain and supply chain. And also forges a bridge and link for mutual benefit and win-win situation of the countries along the route. Therefore, countries along the route should work together to promote the construction of regional lithium trade corridors, and strive to realize the effective connection of railroad and highway logistics networks of the countries along the route, and positively build an all-round, multi-level and composite logistics network of the countries along the route.

**Comments 7:** Finally, the paper is poorly written and this current form requires serious improvement in all sections.

**Response 7：**Thank you very much for reviewing our manuscript. We value your comments and want to improve our work. We apologize for your comment that the paper was poorly written. We will endeavor to raise the standard of our work to ensure that future manuscripts are of a higher quality. If you have any specific suggestions, we would be very grateful if you could provide them. This will help us to make better improvements in the future.

**Response to Reviewer #2’s comments:**

**Comments 1:** Analysis of the evolution of trade import and export patterns should prioritize the overall spatial characteristics rather than merely discussing the network status of nodes and the strength of their connections. Additionally, assertions regarding changes in these patterns should be supported with literature or empirical evidence rather than being based on subjective speculation.

**Response 1:** We completely agree with your comment. Firstly, in chapter 3, this study firstly describes the overall import and export trade patterns of the lithium trade network separately. Secondly, in the chapter 4, network characterization section, we changed the layout of the network pattern characterization section, and preferentially considered the overall spatial characteristics of the lithium trade network by using the network density, core-periphery structure, and block model. Block model is a new addition to study the overall spatial characteristics. Then it is about the network position of the nodes and the strength of their connections. Finally, assertions about changes in these patterns are based on the results of empirical analysis. Changes have been made in some sections that resemble subjective speculations, which we have annotated in the text, marking them in blue. Considering the changes are too numerous to list in a reply letter, please see the blue highlighted sections in the revised manuscript.

**Comments 2:** Analysis of structural features should focus on elucidating the relationships among different methodologies and the scientific questions they address, rather than merely supplementing the workload with classical social network analysis techniques.

**Response 2：**We completely agree with your comment. According to your suggestion, we have reorganized the layout of the research methodology and the results of the corresponding section of the analysis, and we have added to the text the relationships between them and the problems they address. The following is an explanation of the additions we made to the research methods section of the text:

In this paper, we first use network density and trade links in social network analysis to present the overall characteristics of the Belt and Road lithium trade network, and measure the node strength through the core-edge structure, block model, centrality and PageRank algorithm to portray the characteristics of different nodes in the trade network. Secondly, the QAP method is applied to explore the factors affecting the evolution of the lithium trade pattern of the Belt and Road in five dimensions, including the level of economic development, institutional factors, scientific and technological level, geography and cultural factors. It tries to provide theoretical support and policy suggestions for the development and cooperation of lithium resources market of the countries along the Belt and Road in the context of unilateral protectionism and geopolitical conflicts in today’s world.

**Comments 3:** Although numerous methods are employed throughout this paper to meet varied research objectives, it is advisable to consolidate these methods and present them in Chapter Two. This arrangement will clarify the interrelationships among the methods and their roles within the overarching research aims.

**Response 3：**We completely agree with your comment. According to your suggestion, we have combined and presented these methods in chapter 2. Below is our reorganized order of research methods. Considering the formulae may be messy in the system, we can’t list them in the response letter, please see the revised draft for details.

**2.3 Research methodology**

**2.3.1 Network density and reciprocity**

**2.3.2 Core periphery structure**

**2.3.3 Block model**

**2.3.4 Centrality**

**2.3.5 PageRank algorithm**

**2.3.6 Quadratic assignment procedure method**

**Comments 4:** The selection of factors influencing the evolution of trade patterns in foundational lithium products under the "Belt and Road" initiative needs to be underpinned by additional literature to demonstrate their validity and scientific soundness.

**Response 4：**We completely agree with your comment. According to your suggestion, we have added some additional literature to demonstrate the selection of factors’ validity and scientific soundness. The details are as follows:

**(1) Level of economic development[38]. Given that the level of economic development will have a certain impact on the occurrence of international trade and the volume of trade, the matrix of the difference in the gross domestic product of each economy is selected as an unbiased variable. (2) Institutional factors[13][38][39]. National laws, institutions and trade relations have a direct impact on the development of trade between countries, and scholars are mainly based on the United Nations Global Governance Indicators to examine institutional differences. This paper selects three types of indicators to reflect its impact on trade, firstly, the average tariff rate of mineral products under the HS code, tariff rate is the most important expression of international trade policy, is the proportion of the tax amount calculated when taxing the object of taxation as stipulated in the Customs Tariff Rules, which can have a direct impact on the price of lithium imports and exports. Secondly, to examine the impact of political stability and regional trade agreements, political stability is the basis for trade, and regional trade agreements between the two countries to help strengthen the intensity of trade. Therefore, the matrix of regional trade agreements between economies, the absolute value matrix of the difference in political stability and the absolute value matrix of the difference in the average tax rate of mineral products are selected as unbiased variables. (3) Technology level[40]. Technological change in changing a country's energy structure at the same time will trigger the restructuring of the relationship between energy supply and demand, thus creating a new focus of energy geopolitical games. Therefore, this paper selects the patent difference matrix of each country as an unbiased variable. (4) Geographic factors[39]. Geographic distance and bordering is an important factor affecting the occurrence or not of international trade and trade intensity, the closer the geographic distance between two countries the lower the transportation cost will be, making the possibility of trade between the two countries greatly increased.** **(5) Cultural factors[35]. Cultural identity may lead to differences in consumer brand choice and trade levels, and countries with similar cultural backgrounds are more likely to be on the same plate.**

**References:**

[13] Qi W, Li Q. Study on the dynamic evolution characteristics and influencing factors of global new energy vehicle trade network. World Regional Studies. 2023.

[35]	Zhong Z, Qin C. The Trade Network Structure of "One Belt One Road" and its Influence Factors: A Study Based on Analytic Network Process. International Economics and Trade Research. 2017;33(05):16-28.

[38]	Cong H, Zou D, Gao B, Shao J. Network Patterns and Influence Factors of New Energy Vehicle Trade Along the Countries of the Belt and Road. Economic Geography. 2021;41(7):109-18.

[39]	Jiang H. Research on the Pattern and Influencing Factors of China's Grain International Trade Based on Social Network: Jilin University; 2021.

[40]	Wu A, Zhang X, Song X, Li R. Network Structure of Global Nuclear Power Equipment Trade and Its Influencing Factors from 2000 to 2019. Economic Geography. 2022;42(07):126-34+94.

**Comments 5:** The policy recommendations provided in the document should align more closely with the analytical results. The current analysis is overly generalized and lacks a strong connection to the substantive content of the research.

**Response 5：**We completely agree with your comment. According to your suggestion, we have added an extra section in the conclusion section, which gives relevant countermeasure recommendations for the Belt and Road Initiative countries (China) and the Belt and Road participating countries respectively. The details are as follows:

6.2 Suggestions

6.2.1 Suggestions for Belt and One Road Initiative country (China)

(1) Grasp the development opportunities of the Belt and Road Initiative, China should actively discuss and promote the establishment of a strategic cooperation mechanism for lithium resources in countries along the Belt and Road Initiative. The lithium resources governance system is in the early stages of development, with no governance core and authority, and a lack of global coordination mechanisms. China should actively promote the coordination of interests between resource countries and consumer countries, establish lithium industry chain organizations, promote the formation of a global consensus, and put forward a global program for the sustainable supply of green lithium resources.

(2) China should strengthen the degree of domestic lithium resources exploration and development, strengthen the technological innovation of lithium extraction from salt lakes, and increase the investment in the search for lithium salt lakes, lithium pyroxene and lithium mica. Meanwhile, we should also actively build a safe, stable and diversified supply system of lithium resources along the Belt and Road. Strengthen diplomatic relations with the South American “lithium triangle”, enhance the level of strategic cooperation, the use of high-level diplomatic means to create a favorable international investment environment, technology and industrial investment in exchange for local lithium resources. Enhance China's lithium resources in the domestic and international double cycle the safety and security capacity of China's lithium resources in the domestic and international double cycle.

(3) China should improve its foreign-related legal service system. Enterprises not only have to consider industrial, technological, financial and other aspects of risk factors, but also face the host country as well as other countries of the political and social risk of the test. In order to effectively protect the safety of private enterprises investment in overseas minerals, China needs to establish a sound international commercial tribunal and joint arbitration mechanism to provide a trustworthy and dependable dispute resolution platform for China's overseas investment entities. Meanwhile, it should help enterprises carry out compliance training for overseas investment and enhance the comprehensive competitiveness of Chinese enterprises.

6.2.2 Suggestions for Belt and Road participating countries (other countries)

(1) Core countries such as Chile, Argentina and Russia should actively promote the extension of their export markets to Southeast Asia, South Asia, Eastern Europe and Africa, so as to promote the realization of mutual benefits and interconnection of the Belt and Road lithium trade network. Core countries and edge countries should maintain stable relations with the original trading countries on the basis of active development of trade with other countries, with more countries to negotiate free trade agreements, investment protection agreements, reduce trade costs and improve the stability of the network.

(2) Belt and Road countries need to work together with the initiative country (China) to study the creation of rules, enhance the Belt and Road Initiative's global issue-setting and rule-making capabilities, and promote the transformation of the Belt and Road Initiative from commodity-factor flow-type opening to rule system-type opening. The co-construction countries will strengthen the construction of multilateral cooperation platforms for lithium resources, and enhance inter-regional cooperation around the Middle East, Asia-Pacific, Russia-Central Asia, Latin America, Africa and other regions according to their resource endowments.

(3) Actively participate in the construction of “Six Corridors, Six Roads, Multiple Countries and Multiple Ports” to improve the logistics accessibility of the countries along the routes, and build a modern comprehensive transportation system. Poland and Hungary, as the important gateways for China-EU liner to enter the European Union, should grasp the role as important nodes in the transportation network of the Belt and Road, and deepen economic and trade cooperation with China and other Asian countries. As an important carrier of the “Belt and Road” connectivity, China-EU liner maintains the stability and smoothness of the international industrial chain and supply chain. And also forges a bridge and link for mutual benefit and win-win situation of the countries along the route. Therefore, countries along the route should work together to promote the construction of regional lithium trade corridors, and strive to realize the effective connection of railroad and highway logistics networks of the countries along the route, and positively build an all-round, multi-level and composite logistics network of the countries along the route.

**Comments 6:** The introduction of the paper should be significantly enhanced with additional literature, addressing topics including but not limited to the global trade of lithium products and the application of social network analysis methodologies.

**Response 6：**We completely agree with your comment. According to your suggestion, we have added some additional literature. The details are as following and the references are in revised manuscript:

In 2013, Chinese President Xi Jinping proposed the Belt and Road Initiative (BRI**)**; and in 2023, the third Belt and Road Summit on International Cooperation has been held in Beijing. The Belt and Road Cooperation Initiative, a model of South-South cooperation, has produced impressive results in the last ten years in the fields of energy, commerce and economy, building infrastructure, and green development. It has also developed into a significant global public utility and useful platform that has aided in global development and has kept moving in the direction of sustainability, high standards, and improving people's quality of life[1]. In light of the intricate and significant changes occurring in the world today, including the escalation of protectionist inclinations and the persistent rise in uncertainty regarding economic globalization, China has offered a Chinese solution to the global governance system by fostering greater collaboration among the vast majority of developing nations, advancing the economic growth and infrastructure building of the nations along the Belt and Road, and fostering friendship and prosperity. Considering the intricate and significant changes taking place in the contemporary world, the BRI is a major initiative for China to integrate into the Eurasian continent in the new era, and the pattern of energy power shift that characterizes the Belt and Road region is destined to raise major issues[2]. The unstable pattern of international trade and the uneven distribution of key mineral resources resulted in a complex trading dynamic between the nations involved in the Belt and Road, which is not amenable to easy definition and description, forming a complex network structure[3]. In addition, the trading network has a "hybrid" structural framework that exhibits a link design consisting of both hub-and-spoke and fully connected configurations, resulting in high levels of vulnerability and robustness[4].

Regarding social network research, Freeman[5] systematically described the history of the development of social network analysis methods, and argued that many of the concepts in social network theory originated in graph theory, where a set of points consisting of many points and their corresponding lines or edges connecting the points to each other can be depicted on a blank piece of graph paper. Subsequently, Newman[6] found that networks are usually not random and that they have very distinct statistical features, some of which, such as high clustering coefficients and highly skewed degree distributions, are common in networks of all types. Serrano[7] found that scale-free distributions, small-world properties, and degree-correlation features between different vertices make the WTN a complex network, far from being well described by the classical stochastic network. Garlaschelli[8] first defined the world trade network as a network defined by trade relations among countries in the world. Fagiolo[9] used a weighted network approach to study the empirical properties of the network of trade relations among countries and its evolution over time, and found that most countries have weak trade links, but some countries have strong links. Alexandre[10] used a link prediction algorithm based on a current snapshot of the network aimed at predicting its future evolution, and applied it to the international trade data among countries to obtain better results. Ashadun Nobi et al. constructed a minimum spanning tree in world trade based on cross-correlations between time series of different commodities and found that China's influence on the trade network began to emerge after 2004[11]. In recent years, the social network analysis method is widely used in international trade and other fields, and the material, information and spatial flows of international trade are one of the main research contents of world economic geography. Scholars at home and abroad apply this method in the study of trade networks in different fields and commodities, such as Guo Weidong[12] studied the evolution of the spatio-temporal pattern and driving mechanism of global military science and technology trade in 3 aspects, namely, product structure, network pattern and influence mechanism. Qi Wei et al.[13] selected the 2017-2020 global new energy automobile trade data as a sample to study the dynamic evolution characteristics of the trade network. Xiaoliang Jia et al.[14] proposed a novel wavelet-based complex network method, aim to study the evolution characteristics of the integration and diversification for the world's crude oil market, from the structural perspective of global oil prices’ interdependence. He Shengbing et al.[15] used the 54 environmental products’ trade data of the “Belt and Road” countries from 2000 to 2020 to constructed an environmental product trade network, and used the time-indexed random graph model to explore the structural characteristics of the regional environmental product trade network and its evolution mechanism. In addition, some scholars have also utilized social network theory to construct the trade network of nickel ore[16], chromium ore[17] and licorice extract[18] to analyze their trade structure and trade competitiveness.

**Regarding the research on lithium, lithium is the lightest metal in nature, with excellent performance, characterized by strong ability to gain and lose electrons and high specific capacity, which is widely used in a number of new technology industries and fields, such as high-energy batteries, energy storage, aerospace, controlled nuclear reactions, and so on[19-20]. Currently, the status of lithium in the international arena is rising, and major economies have increased the importance of mineral resources such as lithium. In 2009, the Japanese government first released the Rare Metals Safeguard Strategy[21]; in 2016, China released the National Mineral Resources Plan (2016-2020), which lists 24 minerals (groups) such as oil, natural gas, and other minerals as strategic minerals[23] ; in 2022, the U.S. Geological Survey published a new list of 50 key minerals, and the Australian government released the 2022 Key Minerals Strategy, with lithium included in the above strategic list[22,24]. However, lithium has a certain economic vulnerability, and its industrial chain can be mainly divided into four levels, upstream products (lithium raw ore), basic lithium products, deep-processed lithium products and downstream products[25]. In recent years, the social network analysis method has also been introduced in lithium resources and products trade research. Chen Guang[26] analyzed the characteristics of global lithium carbonate and lithium hydroxide trade communities’ evolution. Zhu Lili et al.[27-28] analyzed the control power of China in the international trade network of lithium hydroxide and lithium carbonate. Wu Qiaosheng et al.[29] established a global dynamic trade network for lithium products, analyzing the degree of trade diversity and trade influence of China in the global trade network through weighted degree and other indicators. However, most of the above studies focus on the community division of lithium trade or the controlling role of a certain country in the trade network from the global or China’s perspective, and less on the lithium trade pattern evolution and its influencing factors from the Belt and Road perspective, which makes it difficult for the countries along the route to study their relative position and influence in the lithium trade network.**

**In conclusion, there are currently more abundant international studies related to lithium, and there are also many studies on trade networks. However, some studies have utilized the intensity of access to identify important nodes, but have not taken into account the importance of the nodes associated with them in the trade network. In view of this, this study not only portrays and analyzes the long time series dynamic network evolution of lithium in the countries along the Belt and Road and its influencing factors, but also identifies the key nodes in the trade network of the Belt and Road by using the PageRank (PR) algorithm. The PR algorithm is used to identify key nodes in the “Belt and Road” trade network, and the PR algorithm can rank the search results to prioritize the more important and core web pages or nodes[30]. Unlike the existing literature that uses access degree to identify important nodes in the network, this study attempts to combine the centrality, core-edge structure, and more advanced PR algorithms to explore the evolution of the spatio-temporal pattern of lithium trade in the perspective of the Belt and Road, to analyze the key nodes in the network, and to identify the key nodes in the “Belt and Road” trade network among the countries along the routes. The role and status of the countries along the route in the “Belt and Road” lithium trade network, in order to provide a scientific basis for the selection of trade partners of the countries along the route. It also analyzes the influencing factors of the trade network evolution from five perspectives: economy, system, science and technology, geography and culture, with a view to optimizing the energy trade strategies and policies under the framework of “Belt and Road”, and guaranteeing the high-quality development of China's new energy and new technology industries.**

**Response to Reviewer #3’s comments:**

**Comments 1:** What are the main indicators of social network analysis that need to be introduced in the Research Methodology section? What is the reason why the paper only selected some indicators? What is the reason why the author did not perform block modeling and core edge analysis?

**Response 1：**Thank you very much for your comment. The main indicators of social network analysis that this paper would like to introduce in the research method section are network density, core periphery structure, centrality, and quadratic assignment procedure methods. The reasons for choosing these methods are as follows: this study uses network density, trade links, core periphery structure, and block model in social network analysis to present the overall characteristics of the Belt and Road Lithium trade network, and measures the node strength through centrality and PR algorithms to portray the characteristics of different nodes in the trade network. Some influencing factors of this trade network are analyzed by using the quadratic assignment procedure method. In part 4.3, we did the core periphery structure analysis and tried to do the block model analysis as follows:

**4.3 Block model analysis**

**Hierarchical clustering was performed through the CONCOR function in the UCINET software, selecting 2 and 0.2 as the maximum segmentation depth and convergence criteria, with a maximum of 25 iterations, and the network was divided into four blocks, as shown in Table 3.**

**Table 3. Characteristics of trade relations in each section of the trade network**

| **Year** | **Block** | $I_{k}/I_{kt}$ | $I_{ke}/I_{ki}$ | $\left( n_{k}-1 \right)/\left( n-1 \right)$ | **Classification** |
| --- | --- | --- | --- | --- | --- |
| **2000** | **1** | **42.05%** | **15.00%** | **4.26%** | **Export trade sector** |
|  | **2** | **44.32%** | **35.00%** | **6.38%** | **Two-way trade sector** |
|  | **3** | **7.95%** | **3.33%** | **0.00%** | **Isolated marginal sector** |
|  | **4** | **5.68%** | **46.67%** | **8.51%** | **Import trade sector** |
| **2003** | **1** | **15.38%** | **68.57%** | **3.77%** | **Export trade sector** |
|  | **2** | **15.38%** | **10.48%** | **75.47%** | **Isolated marginal sector** |
|  | **3** | **23.08%** | **4.76%** | **1.89%** | **Export trade sector** |
|  | **4** | **46.15%** | **16.19%** | **56.60%** | **Isolated marginal sector** |
| **2008** | **1** | **51.06%** | **69.33%** | **53.33%** | **Import trade sector** |
|  | **2** | **19.15%** | **2.67%** | **46.67%** | **Isolated marginal sector** |
|  | **3** | **27.66%** | **18.67%** | **25.00%** | **Export trade sector** |
|  | **4** | **2.13%** | **9.33%** | **10.00%** | **Isolated marginal sector** |
| **2013** | **1** | **65.06%** | **60.81%** | **37.14%** | **Two-way trade sector** |
|  | **2** | **13.25%** | **9.46%** | **32.86%** | **Isolated marginal sector** |
|  | **3** | **12.05%** | **10.81%** | **2.86%** | **Export trade sector** |
|  | **4** | **9.64%** | **18.92%** | **24.29%** | **Isolated marginal sector** |
| **2019** | **1** | **56.00%** | **75.96%** | **37.50%** | **Two-way trade sector** |
|  | **2** | **24.00%** | **19.23%** | **39.06%** | **Isolated marginal sector** |
|  | **3** | **20.00%** | **4.81%** | **14.06%** | **Export trade sector** |
|  | **4** | **2.13%** | **0.00%** | **4.69%** | **Isolated marginal sector** |
| **2022** | **1** | **51.49%** | **33.66%** | **34.62%** | **Two-way trade sector** |
|  | **2** | **14.85%** | **7.92%** | **13.46%** | **Export trade sector** |
|  | **3** | **30.69%** | **29.70%** | **26.92%** | **Two-way trade sector** |
|  | **4** | **2.97%** | **28.71%** | **19.23%** | **Import trade sector** |

**In 2000, the first, second, third and fourth segments were the export trade sector, the two-way trade sector, the isolated marginal sector and the import trade sector, respectively. Among them, the first segment is mainly in Argentina, South Korea, Belarus and other South America, East Asia and Eastern Europe, and the proportion of the internal share of the whole is 42.05%, which is larger than the expected value of 4.26%, classifying it as the export trade segment. The segment is active in foreign export trade. The second segment includes 18 countries such as Russia, Italy, and Malaysia, mainly in Southeast Asia and Eastern Europe. The internal share of the overall is 44.32%, which is greater than the expected value of 6.38%, and the export trade is 35.00% of imports, which classifies it as a two-way trade segment. The third segment includes five countries, including Austria and Slovenia, and exports are 0.00% of imports, which is less than 1, so it is classified as an isolated marginal segment, with inactive trade to internal and external section. The fourth sector is 12 countries such as Chile, China, Kenya, etc., and the internal share of the overall share is 5.68%, which is smaller than the expected value of 8.51%, so it is classified as an import trade sector. The internal trade links of the sector are strong, and the external trade links are weak. From 2003 to 2022, after a long period of trade development, the division of internal plate varies greatly. This is due to the increasing number of countries participating in the B&R trade network, and the internal and external divisions’ segments became gradual complicated. In 2019, the first sector is a bidirectional trade sector, which includes 25 countries such as Argentina, China, Chile, South Korea, Turkey, Singapore, etc., and the second, fourth sector is an isolated edge structure, totaling 40 countries. Two isolated edge sectors appear in both 2013 and 2019, mainly in Central Asia and Africa, such as Pakistan, Tanzania, and Zimbabwe. In 2022, two two-way trade sectors appear for the first time in that year, which have 20 and 15 countries respectively, mostly in the 2019 two-way trade section. Meanwhile, there is no isolated marginal section in that year, reflecting the gradual formation of the lithium trade pattern in the regions along the B&R.**

**Comments 2:** The Research methodology section also needs to introduce the principles and advantages of QAP regression. Suggestions 5.1 and 5.2 are placed in the research methods section.

**Response 2：**We completely agree with your comment. According to your suggestion, we refer to Liu Jun‘s book《Lectures on Whole Network Approach: A Practical Guide to UCINET》and Krackhardt's article, adding an introduction on the principles and advantages of QAP regression in section 2.3 of the research methodology. We put part 5.1 in part 2.3, i.e., 2.3.6 in the revised draft. Considering that the model construction and indicator selection in part 5.2 is too long, it would be too lengthy if we put it in part 2, so we put it in part 5. The details are as follows:

**2.3.6 Quadratic assignment procedure method**

**The QAP method, or Quadratic Assignment Procedure, captures the correlation and regression between relational data. It is based on the substitution of matrix data and gives the correlation coefficients between two matrices by comparing the grid values corresponding to each square matrix, while the coefficients are subjected to nonparametric tests[36]. QAP regression, on the other hand, examines the regression relationship between multiple factors or matrices and a matrix, and evaluates the significance of the coefficient of determination**$R^{2}$**, which is a good method that can rule out spurious structural relationships. When performing QAP multivariate regression, all matrices must be N x N square matrices. This approach reduces the problem of a multivariate partial regression coefficient to a simple regression where the bias of the regression analysis does not increase with autocorrelation. In this bivariate form, QAP can assess whether the correlation is significantly different from zero and whether the corresponding multiple regression is significantly different from zero[37].**

**References:**

[36] Liu J. Lectures on Whole Network Approach: A Practical Guide to UCINET, Shanghai People's Publishing House and Gezhi Press; 2009.

[37] Krackhardt D. Predicting with networks: Nonparametric multiple regression analysis of dyadic data. Social Networks. 1988;10(4):359-81.

**Comments 3:** Section 3-4 needs to focus on the role of China and the "the Belt and Road" policy in the lithium trade network.

**Response 3：**Thank you for your comment. we try to add the role of China and the “Belt and Road” policy in the lithium trade network, which can be seen in Section 5, where we focus on the role of China and the “Belt and Road” policy in the lithium trade network in the analysis of factors affecting the lithium trade network. We focus on the role of China and the “Belt and Road” policy in the lithium trade network in the analysis of factors affecting the lithium trade network in section 5, that is, the institutional factors selected the existence of regional trade agreements between economies as variables. Regional Trade Agreement (RTA) refers to an international treaty concluded between two or more countries or different tariff regions in order to eliminate various trade barriers between members and standardize the trade and cooperation relationship between them, which can reflect the signing of RTAs between countries along the Belt and Road, as well as the ability to promote lithium-related trade between countries along the Belt and Road. countries to conduct lithium-related trade. Secondly, section 3-4 mainly analyze the pattern of lithium trade network and its overall characteristics. The evolution of the pattern is objective and based on data and empirical analysis, and in the expression of the change of its characteristics, we also pay attention to the role of China and the “Belt and Road” policy in the lithium trade network, for example, in the analysis of the overall change of the trade pattern and centrality.

**Comments 4:** It is necessary to supplement the theoretical basis for the indicators selected in section 5.2.

**Response 4：**We completely agree with your comment. According to your suggestion, in section 5.2, we have added some supplement of the theoretical basis for indicators election. The details are as follows:

**(1) Level of economic development[38]. Given that the level of economic development will have a certain impact on the occurrence of international trade and the volume of trade, the matrix of the difference in the gross domestic product of each economy is selected as an unbiased variable. (2) Institutional factors[13,38-39]. National laws, institutions and trade relations have a direct impact on the development of trade between countries, and scholars are mainly based on the United Nations Global Governance Indicators to examine institutional differences. This paper selects three types of indicators to reflect its impact on trade, firstly, the average tariff rate of mineral products under the HS code, tariff rate is the most important expression of international trade policy, is the proportion of the tax amount calculated when taxing the object of taxation as stipulated in the Customs Tariff Rules, which can have a direct impact on the price of lithium imports and exports. Secondly, to examine the impact of political stability and regional trade agreements, political stability is the basis for trade, and regional trade agreements between the two countries to help strengthen the intensity of trade. Therefore, the matrix of regional trade agreements between economies, the absolute value matrix of the difference in political stability and the absolute value matrix of the difference in the average tax rate of mineral products are selected as unbiased variables. (3) Technology level[40]. Technological change in changing a country's energy structure at the same time will trigger the restructuring of the relationship between energy supply and demand, thus creating a new focus of energy geopolitical games. Therefore, this paper selects the patent difference matrix of each country as an unbiased variable. (4) Geographic factors[39]. Geographic distance and bordering is an important factor affecting the occurrence or not of international trade and trade intensity, the closer the geographic distance between two countries the lower the transportation cost will be, making the possibility of trade between the two countries greatly increased. (5) Cultural factors[35]. Cultural identity may lead to differences in consumer brand choice and trade levels, and countries with similar cultural backgrounds are more likely to be on the same plate.**

**References:**

[13] Qi W, Li Q. Study on the dynamic evolution characteristics and influencing factors of global new energy vehicle trade network. World Regional Studies. 2023.

[35]	Zhong Z, Qin C. The Trade Network Structure of "One Belt One Road" and its Influence Factors: A Study Based on Analytic Network Process. International Economics and Trade Research. 2017;33(05):16-28.

[38]	Cong H, Zou D, Gao B, Shao J. Network Patterns and Influence Factors of New Energy Vehicle Trade Along the Countries of the Belt and Road. Economic Geography. 2021;41(7):109-18.

[39]	Jiang H. Research on the Pattern and Influencing Factors of China's Grain International Trade Based on Social Network: Jilin University; 2021.

[40]	Wu A, Zhang X, Song X, Li R. Network Structure of Global Nuclear Power Equipment Trade and Its Influencing Factors from 2000 to 2019. Economic Geography. 2022;42(07):126-34+94.

**Comments 5:** Policy recommendations should be separately listed as a section of content. In addition, countermeasures and suggestions should be given to the Belt and Road Initiative (China) and participating countries (other countries) respectively.

**Response 5：**We completely agree your comment. According to your suggestion, we have added an extra section in the conclusion section, which gives relevant countermeasures and suggestions for the Belt and Road Initiative countries (China) and the Belt and Road participating countries (other countries) respectively. The details are as follows:

6.2 Suggestions

6.2.1 Suggestions for Belt and One Road Initiative country (China)

(1) Grasp the development opportunities of the Belt and Road Initiative, China should actively discuss and promote the establishment of a strategic cooperation mechanism for lithium resources in countries along the Belt and Road Initiative. The lithium resources governance system is in the early stages of development, with no governance core and authority, and a lack of global coordination mechanisms. China should actively promote the coordination of interests between resource countries and consumer countries, establish lithium industry chain organizations, promote the formation of a global consensus, and put forward a global program for the sustainable supply of green lithium resources.

(2) China should strengthen the degree of domestic lithium resources exploration and development, strengthen the technological innovation of lithium extraction from salt lakes, and increase the investment in the search for lithium salt lakes, lithium pyroxene and lithium mica. Meanwhile, we should also actively build a safe, stable and diversified supply system of lithium resources along the Belt and Road. Strengthen diplomatic relations with the South American “lithium triangle”, enhance the level of strategic cooperation, the use of high-level diplomatic means to create a favorable international investment environment, technology and industrial investment in exchange for local lithium resources. Enhance China's lithium resources in the domestic and international double cycle the safety and security capacity of China's lithium resources in the domestic and international double cycle.

(3) China should improve its foreign-related legal service system. Enterprises not only have to consider industrial, technological, financial and other aspects of risk factors, but also face the host country as well as other countries of the political and social risk of the test. In order to effectively protect the safety of private enterprises investment in overseas minerals, China needs to establish a sound international commercial tribunal and joint arbitration mechanism to provide a trustworthy and dependable dispute resolution platform for China's overseas investment entities. Meanwhile, it should help enterprises carry out compliance training for overseas investment and enhance the comprehensive competitiveness of Chinese enterprises.

6.2.2 Suggestions for Belt and Road participating countries (other countries)

(1) Core countries such as Chile, Argentina and Russia should actively promote the extension of their export markets to Southeast Asia, South Asia, Eastern Europe and Africa, so as to promote the realization of mutual benefits and interconnection of the Belt and Road lithium trade network. Core countries and edge countries should maintain stable relations with the original trading countries on the basis of active development of trade with other countries, with more countries to negotiate free trade agreements, investment protection agreements, reduce trade costs and improve the stability of the network.

(2) Belt and Road countries need to work together with the initiative country (China) to study the creation of rules, enhance the Belt and Road Initiative's global issue-setting and rule-making capabilities, and promote the transformation of the Belt and Road Initiative from commodity-factor flow-type opening to rule system-type opening. The co-construction countries will strengthen the construction of multilateral cooperation platforms for lithium resources, and enhance inter-regional cooperation around the Middle East, Asia-Pacific, Russia-Central Asia, Latin America, Africa and other regions according to their resource endowments.

(3) Actively participate in the construction of “Six Corridors, Six Roads, Multiple Countries and Multiple Ports” to improve the logistics accessibility of the countries along the routes, and build a modern comprehensive transportation system. Poland and Hungary, as the important gateways for China-EU liner to enter the European Union, should grasp the role as important nodes in the transportation network of the Belt and Road, and deepen economic and trade cooperation with China and other Asian countries. As an important carrier of the “Belt and Road” connectivity, China-EU liner maintains the stability and smoothness of the international industrial chain and supply chain. And also forges a bridge and link for mutual benefit and win-win situation of the countries along the route. Therefore, countries along the route should work together to promote the construction of regional lithium trade corridors, and strive to realize the effective connection of railroad and highway logistics networks of the countries along the route, and positively build an all-round, multi-level and composite logistics network of the countries along the route.
